# Clinical and phantom validation of a deep learning based denoising algorithm for F-18-FDG PET images from lower detection counting in comparison with the standard acquisition

**DOI:** 10.1186/s40658-022-00465-z

**Published:** 2022-05-11

**Authors:** Gerald Bonardel, Axel Dupont, Pierre Decazes, Mathieu Queneau, Romain Modzelewski, Jeremy Coulot, Nicolas Le Calvez, Sébastien Hapdey

**Affiliations:** 1 Nuclear Medicine, Centre Cardiologique du Nord, Saint-Denis, France; 2 Nuclear Medicine, Hopital Delafontaine, Saint-Denis, France; 3 Esprimed SAS, Villejuif, France; 4 Nuclear Medicine Department, Henri Becquerel Cancer Center, Rouen, France; 5 QuantIF-LITIS EA4108, Rouen University Hospital, Rouen, France

**Keywords:** PET/CT, Deep-learning, Denoising

## Abstract

**Background:**

PET/CT image quality is directly influenced by the F-18-FDG injected activity. The higher the injected activity, the less noise in the reconstructed images but the more radioactive staff exposition. A new FDA cleared software has been introduced to obtain clinical PET images, acquired at 25% of the count statistics considering US practices. Our aim is to determine the limits of a deep learning based denoising algorithm (SubtlePET) applied to statistically reduced PET raw data from 3 different last generation PET scanners in comparison to the regular acquisition in phantom and patients, considering the European guidelines for radiotracer injection activities. Images of low and high contrasted (SBR = 2 and 5) spheres of the IEC phantom and high contrast (SBR = 5) of micro-spheres of Jaszczak phantom were acquired on 3 different PET devices. 110 patients with different pathologies were included. The data was acquired in list-mode and retrospectively reconstructed with the regular acquisition count statistic (PET100), 50% reduction in counts (PET50) and 66% reduction in counts (PET33). These count reduced images were post-processed with SubtlePET to obtain PET50 + SP and PET33 + SP images. Patient image quality was scored by 2 senior nuclear physicians. Peak-signal-to-Noise and Structural similarity metrics were computed to compare the low count images to regular acquisition (PET100).

**Results:**

SubtlePET reliably denoised the images and maintained the SUV_max_ values in PET50 + SP. SubtlePET enhanced images (PET33 + SP) had slightly increased noise compared to PET100 and could lead to a potential loss of information in terms of lesion detectability. Regarding the patient datasets, the PET100 and PET50 + SP were qualitatively comparable. The SubtlePET algorithm was able to correctly recover the SUV_max_ values of the lesions and maintain a noise level equivalent to full-time images.

**Conclusion:**

Based on our results, SubtlePET is adapted in clinical practice for half-time or half-dose acquisitions based on European recommended injected dose of 3 MBq/kg without diagnostic confidence loss.

**Supplementary Information:**

The online version contains supplementary material available at 10.1186/s40658-022-00465-z.

## Key points

- QUESTION: Is there any interest to use the SubtlePET reconstruction on daily practice low statistics PET images in terms of lesion detectability and quantification.
- PERTINENT FINDINGS: Applied on half-time (or eventually half-dose) acquisitions, SubtlePET correctly recovers the SUV_max_ values of the lesions and maintain a noise level equivalent to full-time images, yielding a lesion contrast-to-noise ratio comparable to that of full statistical PET acquisition based on EU recommended standard of 3 MBq/kg.
- IMPLICATIONS FOR PATIENT CARE: Based on our results, SubtlePET is ready to be used in clinical practice for half-time or half-dose acquisitions. It would offer new perspectives such as, dividing the injected dose by 2, scanning twice as fast when necessary (children, painful patients, long delays in appointments and the need to increase the number of patients per day, emergencies, etc.…) or the possibility to make real whole body and not mid-thigh vertex more easily.

## Introduction

Positron emission tomography coupled with computed tomography (PET/CT) after the injection of F-18-Fluoro-2-deoxy-glucose (F-18-FDG) is a commonly used imaging modality to perform diagnostic and stratify diseases in various pathologies (oncology, cardiology, infectiology, neurology…).

The image quality required to perform an accurate diagnosis is directly influenced by the radiopharmaceutical activity and the acquisition duration. The higher the injected activity or acquisition duration, the less noise in the reconstructed images.

For a given injected activity, increasing the acquisition duration limits the number of patients per day who can benefit from the exam. A longer examination is also less well tolerated by the patient and may increase the risk of motion artifacts. Conversely, for a given acquisition duration, increasing the injected activity leads to higher exposure of the patient and operational dose to the healthcare staff.

Thus, optimizing the use of PET CT technology in a clinical setting needs to balance carefully the injected activity/acquisition duration ratio.

The current standard approach for image reconstruction of PET Raw data is based on iterative methods. The most commonly used being the Ordered Subset Maximum Expectation (OSEM) algorithm, which requires setting up a global number of iterations to reconstruct the image. Theoretically, the higher the number of iterations, the closest to the expected reconstructed image. However, the increased number of iterations generates noise that worsens image quality and might cause misinterpretations and quantification errors by reducing the signal-to-noise ratio.

To reduce PET image noise, different approaches have been proposed. The first approaches incorporate image noise reduction within the reconstruction process. As an alternative to OSEM, the Bayesian Penalized likelihood (BPL) approach, consists in reducing noise between iterations using penalty functions and has been proposed and implemented few years ago on the General Electric PET systems. This algorithm, incorporating a prior in the image distribution, allows the use of a high number of iterations, improving contrast while preventing any dramatic noise increase [1, 2]. However, noise reduction is done at the expense of image quantitation [3].

Other noise reduction approaches consist in the image post-filtering, from the simple Gaussian mean to the more complex non-local means [4], possibly including anatomical priors [5]. Non-stationary approaches, such as multi-scale transform (curvelet or wavelet) [6, 7] have proven to be also of great interest to provide a significant reduction of noise while preserving contrast and important structures [8, 9].

Few years ago, neural networks (NN) organized in multiple layers, called deep learning (DL), have been introduced as an efficient tool to perform image denoising on PET raw data with low statistic. The results, essentially published for brain PET images, suggest that one can recover the same noise level in the images with lower dose than the one obtained with the standard acquisition dose [10–12]. Many NN, from supervised or semi-supervised convolutional neural network (CNN) to unsupervised CNN and their derivatives, have been proposed as post-reconstruction processes [13–15]. More recently, authors proposed to use NN, within the reconstruction framework, to improve PET image quality [16], or to perform directly the image reconstruction from sinograms with a U-NET approach [17].

Essentially developed in the framework of cerebral examinations, few teams have so far been interested in the use of neural networks for denoising in the case of whole body examinations [18, 19]. Based on the work from Stanford University [20], a new FDA and CE cleared software has been introduced (SubtlePET, Subtle Medical, Menlo Park, CA, USA) to improve clinical PET images, on wholebody PET exams acquired at 25% of the count statistics. This NN is based on a 2.5D encoder–decoder U-Net deep convolutional neural network trained on North American datasets and has been evaluated on a small population [21–23]. Only a very recent study investigates the ability of SubtlePET to produce images of adequate diagnostic confidence on 61 patients, considering a counting statistic equivalent to 66% of the original statistic (i.e. a 33% reduction of the original statistic) for old-generation PET scanners (without time of flight-TOF-technology)[24].

According to SubtlePET manufacturer, a 75% dose reduction can be achieved without any information loss with SubtlePET enhanced images, but to our knowledge, nobody has investigated so far, the important topic of model generalizability, i.e., how well can an AI model trained on North American PET data generalize to new patients from different practices, different scanners and different injected dose standards. Therefore, our aim in this study was to assess the performance and potential limitations of SubtlePET on phantom datasets and on a large population of whole body PET clinical exams performed under EANM or EU practice guidelines for FDG injected dose, considering count statistics down to − 66% and on latest generation of PET systems (TOF and SiPM).

## Materials and methods

### Phantom

Three phantom experiments were realized using a NEMA IEC phantom with 3 different acquisition conditions and acquired on our 3 different General Healthcare PET/CT systems (Discovery MI 4 rings, Discovery 710 and Discovery IQ 4 rings).

For the first and second experiments (E1 & E2), the phantom was equipped with a set of 6 fillable spheres (inner diameters/volumes of 10 mm/0.52 mL, 13 mm/1.15 mL, 17 mm/2.57 mL, 22 mm/5.58 mL, 28 mm/11.5 mL, 37 mm/26.5 mL). For E1, 2 syringes of approximately 20 MBq and 10 MBq of F-18-DG (calibrated at the acquisition time) were prepared. The first syringe was injected into the phantom tank filled with water, and the second, diluted on 1 L of water and then used to fill the spheres. This yields a contrast ratio between spheres and background of 5:1 and a background activity of 2 MBq/kg. At the end of E1, approximately 20 MBq of F-18-FDG were reinjected in the phantom background to obtain a contrast ratio of 2:1 (E2).

For the third experiment (E3), the phantom was equipped with a set of 4 micro fillable spheres (inner diameters/volumes of 5.94 mm/31 µL, 6.95 mm/63 µL, 8.23 mm/125 µL, 9.86 mm/250 µL) and the 2 smallest spheres of the standard set mentioned above. The same procedure was applied to reach a final contrast of 5:1.

For each experiment, the phantom was centered in the field-of-view and a list-mode acquisition over one bed-position was performed, allowing the reconstruction of different acquisition durations: the regular clinical duration (PET100), one-half (PET50) and one-third (PET33) and of the regular clinical duration (cf. Table 1). This method allows to simulate a PET tracer dose reduction retrospectively, with resulting simulated low-dose images having equivalent characteristics with PET images actually measured at lower doses [25].

**Table 1:** Clinical acquisition and reconstruction set-up for the 3 PET devices

	DMI4	D710	IQ4
Clinical injected activity	3 MBq/kg	3 MBq/kg	3 MBq/kg
Clinical acquisition time	1.5 min/bed	2 min/bed	2 min/bed
Type of reconstruction	BPL (Q.Clear)	VPFX (OSEM)	BPL (Q.Clear)
Sharp-IR	Yes	Yes	Yes
TOF	Yes	Yes	No
Iterations	–	2	–
Subsets	–	24	–
Regularization parameter	700	–	350
Matrix size	256^2^	256^2^	256^2^
In plane filter	–	6.4 mm	–
Axial filter	–	Heavy	–
Voxel size (mm)	2.73 × 2.73 × 2.79	2.73 × 2.73 × 3.27	2.73 × 2.73 × 3.27

The raw data were reconstructed according to the routinely used OSEM or BPL protocols (cf. Table 1) and for the 1/2 and 1/3 acquisition duration, post-processed with SubtlePET (named PET50 + SP and PET33 + SP).

We used PET/CT images from the first 110 patients who agreed to participate to this study. Those patients benefited from PET examination addressed for various pathologies (oncology or internal medicine representative of the clinical activity) during October 2020. All patients were informed that their data were fully anonymized for research purposes and gave their approval (IRB approval was obtained for this study). PET/CT scans were acquired 60 min after the injection of 3 MBq/kg of F-18-FDG, with an acquisition time varying from 1.5 min/bed position for the DMI4 to 2 min/bed position for D710 and DIQ4 (cf. Table 1). All the PET raw data were natively acquired in the list-mode format, allowing the retrospective reconstruction of lower time/dose-equivalent sinograms. Given the results observed in the phantom experiments regarding the loss of information with PET33 + SP reconstructions, only a subpopulation of 30 patients were reconstructed with a 66% time lowering (PET33) and post-processed with SubtlePET (PET33 + SP) to evaluate the qualitative improvement achieved by SubtlePET. For the whole population, a 50% time lowering was studied (PET50) and enhanced with SubtlePET (PET50 + SP). For the 20 patients with a body mass index (BMI) > 30 kg/m^2^, the SubtlePET algorithm was also applied on the full time acquisition (PET100 + SP) to evaluate the interest of SubtlePET on noisier images. As for the phantom experiments, the reconstruction set-up depends on the PET system used (cf. Table 1).

Except for disease-free patients, one hypermetabolic lesion was delineated on each patient by an experienced nuclear physician. In order to be representative, the choice was made to select different types of lesions: primary or metastatic, small sub-centimetric or larger, homogeneous or heterogeneous, low or high uptake etc.…) from different organs among 60 patients.

### SubtlePET algorithm

SubtlePET uses a 2.5D encoder–decoder U-Net deep convolutional neural network to perform denoising. The software takes a low count PET image (from shorter scan or lower dose) as input and generates a high quality PET image (close to full dose image) as output. It employs a convolutional neural network (CNN)-based method in a pixel’s neighborhood to reduce noise and increase image quality. Using a residual learning approach and optimized for quantitative (L1 norm) as well as structural similarity (SSIM), the software learns to separate and suppress the noise components while preserving and enhancing the structural components.

The networks were trained with paired low- and high-count PET series coming from a wide range of clinical indications and patient BMI and from a large variety of PET/CT and PET/MR devices (10 General Electric, 5 Siemens and 2 Philips models). The training data included millions of paired image patches derived from hundreds of patient scans with multi-slice PET data and data augmentation. All the training PET data was acquired in the USA or Canada with the average injected FDG dose ~ 6 MBq/kg and acquisition time per bed of 2–3 min/bed. For the training regime, low count data was either retrospectively reconstructed or prospectively acquired at 1/4th the acquisition time or dose (i.e., 1.5 MBq/kg at 2–3 min/bed or 6 MBq/kg at 30–45 s/bed).

### Image analysis

#### Quantification

On the phantom experiments, spherical volumes of interest (VOIs) were manually drawn to enclose each visible sphere and on the background (10 cm^3^ spherical VOI located in the central part of the phantom) to measure quantitative parameters. On the patient analysis, the lesions quantitation was measured, using automatic segmentation tools proposed on the AWServer workstation (GE Healthcare, Milwaukee, USA). The background region was proximally defined for each lesion. In addition, a VOI of approximately 6 cm^3^ was also defined on an hepatic healthy region when applicable. For the phantom or the patients, each VOI was perfectly cloned on every sequence (all reconstructions and all acquisition statistic) to get the measurements on the exact same location and prevent any intra-operator variability.

For each VOI, the SUV_max_, SUV_mean_, SUV_peak_ and standard deviation (SD) were recorded to derive: the sphere contrast recovery coefficient (CRC) for the phantom data only, the contrast to noise ratio (CNR) and the background variability (BV) by using:

$${\text{CRC}} = \frac{{\frac{{{\text{SUV}}_{{{\text{max}}}} {\text{ in sphere}}}}{{{\text{SUV}}_{{{\text{mean}}}} {\text{ in backgrcound}}}}}}{{\frac{{\text{Activity concentration in sphere}}}{{\text{Actity concentration in background}}}}};\;{\text{CNR}} = \frac{{\frac{{{\text{SUV}}_{{{\text{max}}}} {\text{ in sphere or lesion}}}}{{{\text{SUV}}_{{{\text{mean}}}} {\text{ in backgrcound}}}}}}{{{\text{SUV}}_{{{\text{SD}}}} {\text{ in Background}}}}\;{\text{and}}\;{\text{BV}} = \frac{{{\text{SUV}}_{{{\text{SD}}}} {\text{ in Background}}}}{{{\text{SUV}}_{{{\text{mean}}}} {\text{ in backgrcound}}}} \times 100$$

We also calculated the percentage variation of SUV_max_, SUV_peak_, CRC, CNR and BV (ΔSUV_max_, ΔSUV_peak_, ΔCRC, ΔCRC and ΔBV respectively) regularly used in clinical practice, between the SubtlePET-enhanced images (PET50 + SP, PET33 + SP, PET100 + SP) and the standard PET100 images.

We studied the correlation between BV and SUV variations (ΔBV and ΔSUV) as a function of the patient BMI to evaluate the efficiency of SubtlePET on patient with noisier images.

Additionally, quantitative image quality metrics like peak signal to noise ratio (PSNR) and structural similarity index (SSIM) were also calculated between the regular duration PET scan (PET100) and the faster PET processed and unprocessed series to assess for the presence or the absence of absolute errors (data loss, corruption, alteration, or exaggeration).

In complement to the quantitative analysis, 2 senior nuclear medicine physicians independently realized a qualitative evaluation of the overall quality of the image, considering a 3 point-scale: (1) insufficient quality for image interpretation; (2) insufficient quality, with noise or heterogeneity but acceptable for interpretation; and (3) image of good quality for optimal interpretation. At the end of their evaluation, in case of disagreement on image quality rating, a joint analysis was performed. Finally, for the PET50 + SP versus PET100 images, the evaluation of quality was summarized by the question: “Would my report have changed considering the PET50 + SP instead of PET100 images?”. To that end, PET100 and PET50 + SP series were presented side by side to each physician independently. All the images were evaluated in one session and there was no waiting period between different images.

### Statistical analysis

We compared the phantom and patient's quantitative data (SUV_max_, SUV_mean_, ΔSUV_max_, ΔSUV_mean_, ΔCRC and ΔCNR) using a Student paired *t* test, with *p* values lower than 0.05 considered as statistically significant.

The comparison of lesion detectability and quality between PET100 and SubtlePET-enhanced images was evaluated by calculating the kappa coefficients for each observer.

All statistical tests were realized with MedCalc 13.1.2.0 and graphs and plots with Excel 2016.

## Results

### Phantom evaluation

**Fig. 1:**
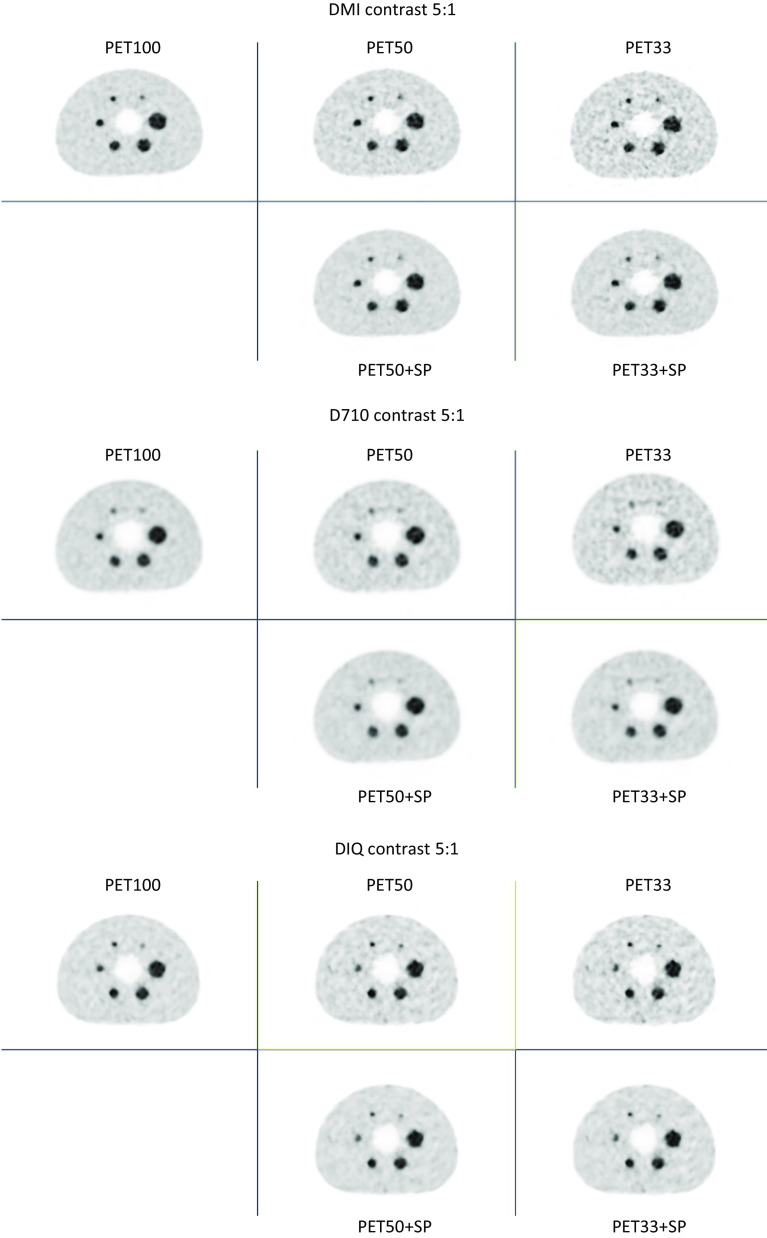
Images from the IEC phantom acquisition for a contrast of 5:1, for each PET systems (Top image: DMI; Middle image: D710 and bottom image: IQ4), acquisition time (1st column: PET100, 2nd colomn: PET50 and 3rd column: PET33) and the application of SubtlePET (2nd row of each image)

**Fig. 2:**
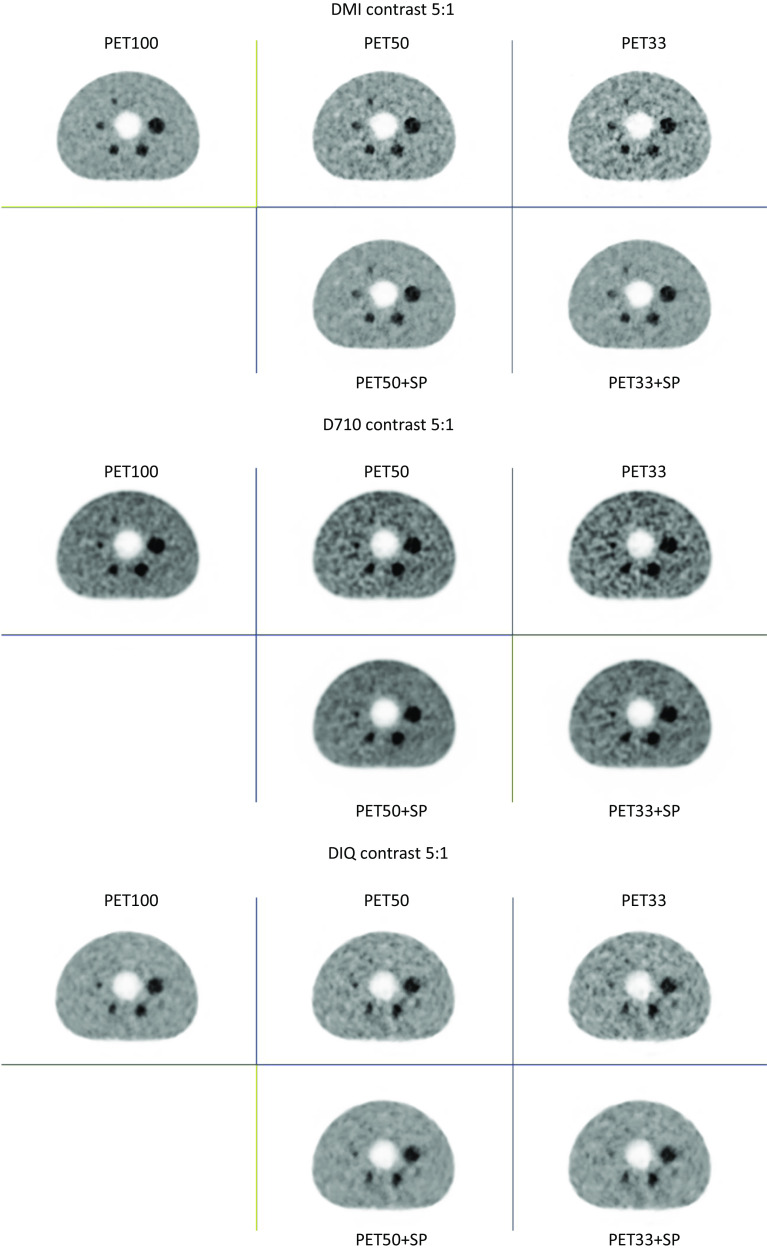
Images from the IEC phantom acquisition for a contrast of 2:1, for each PET system (Top image: DMI; Middle image: D710 and bottom image: IQ4), acquisition time (1st column: PET100, 2nd colomn: PET50 and 3rd column: PET33) and the application of SubtlePET (2nd row of each image)

**Fig. 3:**
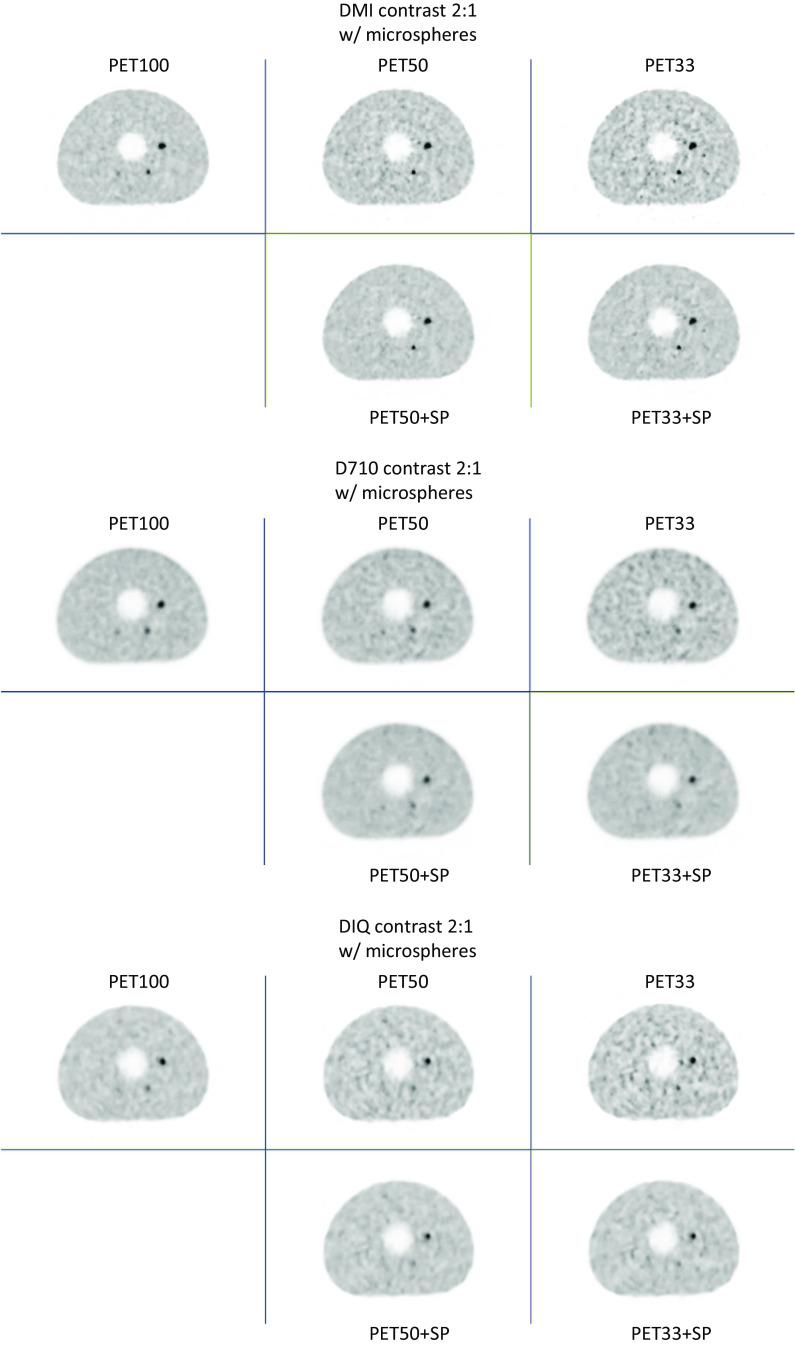
Images from the IEC phantom acquisition equipped with microspheres, for a contrast of 5:1, for each PET system (Top image: DMI; Middle image: D710 and bottom image: IQ4), acquisition time (1st column: PET100, 2nd colomn: PET50 and 3rd column: PET33) and the application of SubtlePET (2nd row of each image)

Figures 1, 2 and 3 present the images of the phantom of experiment E1, E2 and E3, for the 3 PET devices and the 5 reconstructed series (PET100; PET50; PET33; PET50 + SP; PET33 + SP).

These figures clearly show that the noise increases when the acquisition time is reduced. This noise is compensated for by the application of SubtlePET. Table 2 summarizes the improvements in PSNR and SSIM indices in comparison to PET100, considering the slice with all visible spheres from the NEMA IEC phantom experiments (E1 & E2). SubtlePET processing increased the PSNR & SSIM in every instance of PET50 & PET33 images.

**Table 2:** PSNR and SSIM indices variation in comparison to PET100, considering the slice with all visible spheres from the NEMA IEC phantom experiments (E1 & E2)

	PET50	PET50 + SP	PET33	PET33 + SP
*PSNR (E1)*				
D710	41.61	43.07	36.55	37.42
IQ4	38.86	40.30	37.81	40.26
DMI4	38.53	40.19	33.12	33.88
*PSNR (E2)*				
D710	35.60	36.73	35.82	39.29
IQ4	39.27	40.92	34.85	36.98
DMI4	34.71	39.26	30.98	35.04
*SSIM (E1)*				
D710	0.982	0.984	0.972	0.976
IQ4	0.979	0.983	0.968	0.977
DMI4	0.983	0.987	0.968	0.973
*SSIM (E2)*				
D710	0.980	0.981	0.968	0.975
IQ4	0.980	0.983	0.972	0.978
DMI4	0.976	0.980	0.964	0.972

SubtlePET processing increase the PSNR & SSIM in every instance i.e., PET50 & PET33 images

Visually, whatever the experiment and the PET device, SubtlePET seems to reliably denoise and restore the noise level for the PET50 + SP to the level of the PET100 acquisition. For the PET33 acquisition which is much noisier than PET100 and PET50, SubtlePET enhancement (PET33 + SP) significantly reduces the noise but the noise level remained slightly higher than PET100. In terms of detectability, for experiment E1 (standard spheres, contrast S/B = 5), the reduction of the acquisition time (50% or 33%) does not induce any loss of detectability, whatever the PET system. For experiment E2 (standard spheres, contrast S/B = 2), the reduction of the acquisition time induces a loss of detectability for the D710 system (for 50% and 33%) and IQ4 PET system (for 33% only), but not for the DMI4 PET system. Finaly, for experiment E3 (micro spheres), the detectability loss can be observed for the 3 PET systems and 2 acquisitions time (50% and 33%). SubtlePET does not recover the sphere detectability that is not present in the input images, neither for the low contrast (Fig. 2 for D710 PET device), nor for the microspheres (Fig. 3 for the 3 PET systems), whatever the PET system or the acquisition time.

**Fig. 4:**
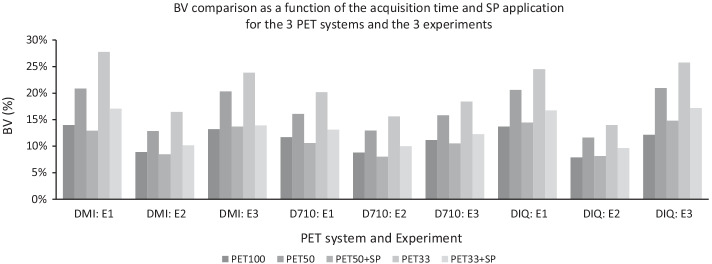
Background variability (BV = SUV standard deviation/SUV mean) as a function of the acquisition time (PET100, PET50 and PET33) and the application of SubtlePET (PET50 + SP and PET33 + SP) for the 3 PET systems (DMI, D710 and IQ4) and the 3 experiments (E1: IEC phantom and contrast 5:1; E2: IEC phantom and contrast 2:1 and E3: IEC phantom w/microspheres and contrast 5:1)

Figure 4 shows the BV as the function of the acquisition time (PET100, PET50 and PET33) and SubtlePET application for the 3 PET systems of experiments E1 and E2. This figure confirms the qualitative observation of Figs. 1 and 2, with a comparable background variability only between PET100 and PET50 + SP series. For the PET33 acquisition, applying the SubtlePET algorithm does not restore correctly the noise level, which remains elevated, whatever the PET system and contrast considered.

Table 3 summarizes the quantitative variation of the spheres quantification. A small but statistically significant SUV_mean_ and SUV_peak_ reduction was observed due to the application of SubtlePET on the PET50 and PET33 data, in comparison with the PET100 data. For the SUV_max_ index, PET50 and PET33 were statistically higher than PET100. When applying the SubtlePET algorithm, the SUV_max_ for PET50 + SP and PET33 + SP became non statistically different from PET100 (mean ΔSUV_max_ = − 4.6 ± 14.2%, *p* = 0.06 and − 1.4 ± 13.6%, *p* = 0.94 for PET50 + SP and PET33 + SP respectively).

**Table 3:** Mean SUV indexes variation in comparison to PET100, considering the visible spheres pooled from the 3 experiments

	PET50	PET50 + SP	PET33	PET33 + SP
*ΔSUV*_*max*_				
Mean value	9.1%	− 4.6%	14.6%	− 1.4%
Standard deviation	17.9%	14.2%	16.5%	13.6%
*p* value	4E−04	0.061	< 1.E4	0.94
*ΔSUV*_*peak*_				
Mean value	1.5%	− 5.0%	2.5%	− 4.7%
Standard deviation	7.8%	7.2%	11.2%	10.2%
*p* value	0.16	5E−04	0.14	0.025
*ΔSUV*_*mean*_				
Mean value	− 0.2%	− 5.7%	0.0%	− 5.5%
Standard deviation	0.3%	8.6%	2.4%	10.7%
*p* value	0.71	< 1.E4	0.96	< 1.E4

p values of the paired *t* test between the SUV values for PET100 and the corresponding SUV for the 4 acquisitions/reconstructions are given

**Fig. 5:**
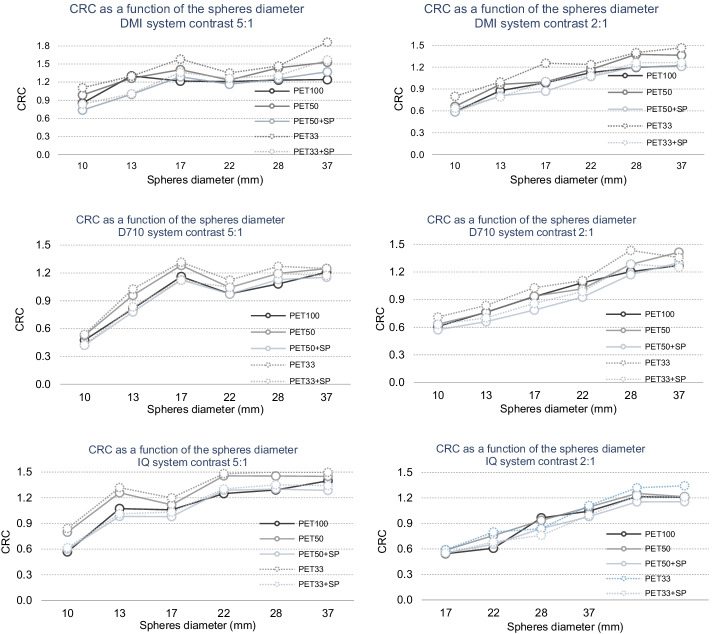
Contrast Recovery (CRC = measured image contrast/theoritical contrast) as a function of sphere diameter, acquisition time (PET100, PET50 and PET33) and SubtlePET application (PET50 + SP and PET33 + SP) for the 3 PET systems (1st row: DMI; 2nd row: D710 and 3rd row: IQ4) and 2 contrasts (1st column:5:1 and 2nd colomn:2:1)

Figure 5 plots the contrast recovery coefficient for each visible sphere as a function of the acquisition for the 3 PET systems (three rows) and contrast (2 columns). Figure 5 illustrates that SubtlePET seems to have a real benefit on contrast recovery, especially on images of contrast 5:1. This is confirmed with the results shown in Table 4, which presents the quantitative variations between the native and SubtlePET–enhanced images for the calculation of the lesion CRC with respect to PET100 acquisition. There was a statistically significant increase of the CRC for the PET50 (10.1 ± 17.4%; *p* < 10^−4^) and PET33 series (15.9 ± 16.1%; *p* < 10^−4^). The application of SubtlePET allows a bias reduction which remains statistically significant for PET50 + SP: − 4.7 ± 13.7% (*p* = 0.02) but not significant for PET33 + SP: − 1.5 ± 12.9% (*p* = 0.87).

**Table 4:** Mean CRC values and CRC variation in comparison to PET100, considering the visible spheres pooled from the 3 experiments

	PET100	PET50	PET50 + SP	PET33	PET33 + SP
Mean (± Std Dev) CRC value	1.02 (± 0.25)	1.11 (± 0.28)	0.98 (± 0.26)	1.19 (± 0.30)	1.03 (± 0.28)
Mean variation (± Std Dev) versus PET100	–	9.3% (± 9,1%)	− 4.1% (± 7.4%)	16.6% (± 12.6%)	0.3% (± 9.3%)
*p* value	–	< 1.E4	0.02	< 1.E4	0.87

*p* values of the paired *t* test between the CRC values for PET100 and the corresponding CRC for the 4 acquisitions/reconstructions are given

**Fig. 6:**
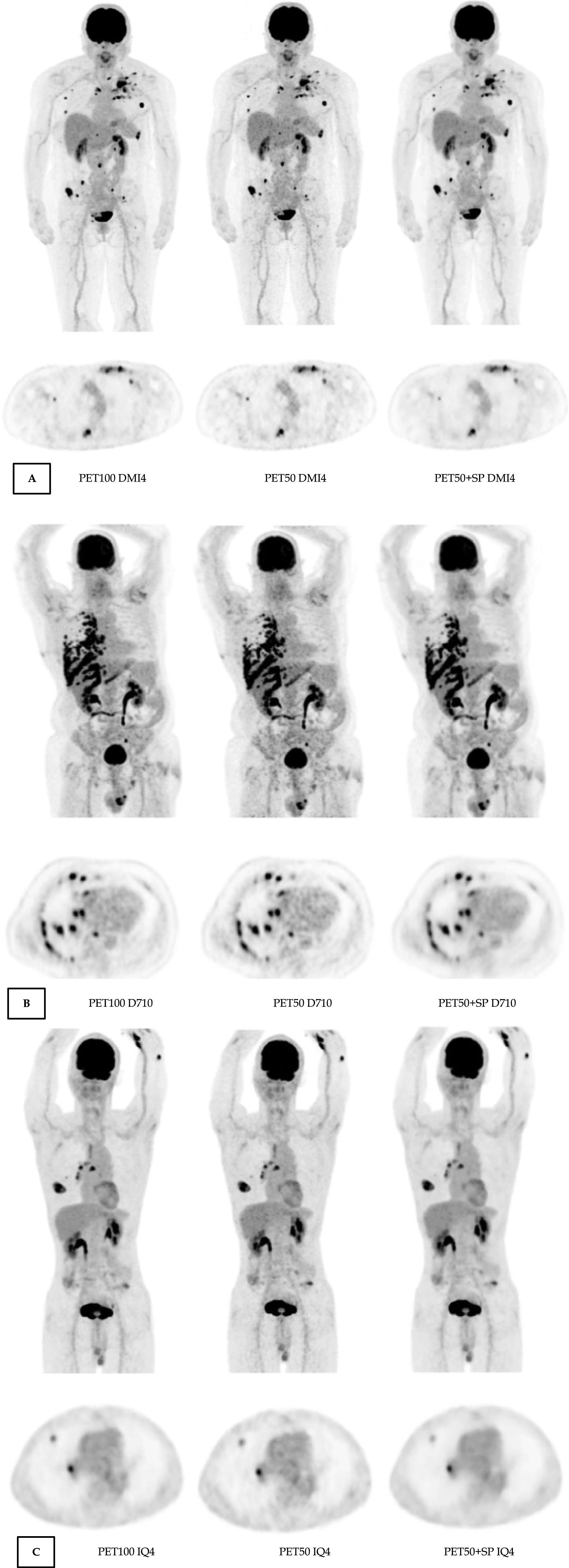
Examples of MIP and axial attenuation corrected PET images reconstructed with PET100, PET50 and PET50 + SP obtained on the DMI4 (**A**), D710 (**B**) and IQ4 (**C**) PET systems. Patients characteristics **A** Female; 60 kg; IBM 26.7; Breast cancer **B** Male; 98 kg; IBM 32.4; Lung cancer; **C** Female; 47 kg; IBM 18.4; Lung cancer;

Based on this whole set of results of the phantom experiments, we decided to limit the application of SubtlePET to the PET50 images for the clinical evaluation, as the best balance between noise control, lesion detectability and quantitative contrast recovery. Figure 6 show exemples of PET100, PET50 and PET50+SP images, for the 3 PET systemes.

### Patients

The clinical indications for the patient studies were largely for cancer diagnoses and follow up, with full demographic information summarized in Table 5.

**Table 5:** Patient and lesion characteristics

Patient	*n* = 110 (%)
*Sex*	
Men	49 (44.5)
Women	61 (55.5)
*Age (mean)*	64 [20–88]
*Pathology*	
Lung cancers	38 (35)
Breast cancers	23 (20)
Head and neck cancers	10 (9)
GastroIntestinal tract cancers	9 (8)
Melanoma	4 (4)
Lymphoma	3 (3)
Sarcoma	1 (1)
Gynecologic cancers	1 (1)
Internal medicine	21 (19)
*Stage*	
Initial	49 (44.5)
Follow-up	61 (55.5)
*Lesion volumes*	
Median	4.8
Minimal	0.2
Maximal	284

#### Performance of SubtlePET in the image quality restoration

Table 6 presents the image quality scoring by the 2 medical physicians. For the PET33 acquisitions, 83.3% of the images were considered non-interpretable due to the high noise level. Applying SubtlePET reduces the rate of non-interpretable images as low as 13.3%. Finally, 26.7% of the PET33 + SP images were considered to be of good quality (0% for PET33 alone). As for the phantom experiments, they remain of lower quality than the PET100 images, which is why we decided not to further analyse the loss of quantification of the PET33(± SP) images of the patient.

**Table 6:** Image quality quotation on a 3-point scale, for the PET 33, PET33 + SP, PET50, PET50 + SP and PET100 images

	PET33	PET33 + SP	PET100	PET50	PET50 + SP	PET100	PET100	PET100 + SP
	30 patients			110 patients			20 patients (BMI > 30)	
Level 1: insufficient quality, interpretation impossible	25 (83.3%)	4 (13.3%)	0 (0%)	18 (16.3%)	0 (0%)	0 (0%)	0 (0%)	0 (0%)
Level 2: insufficient quality but interpretation possible	5 (16.7%)	18 (60%)	5 (16.7%)	90 (81.8%)	17 (15.5%)	16 (14.5%)	8 (40%)	4 (20%)
Level 3: image of good quality	0 (0%)	8 (26.7%)	25(83.3%)	2 (1.8%)	93 (84.5%)	94 (85.5%)	12 (60%)	16 (80%)

For PET50 images, 16.3% were considered of poor quality (level 1) and 81.8% of insufficient quality (level 2). The application of SubtlePET clearly improved all low quality images by making them interpretable (level 1 → level 2) and obtaining a classification of image quality comparable to that of PET100 images.

**Fig. 7:**
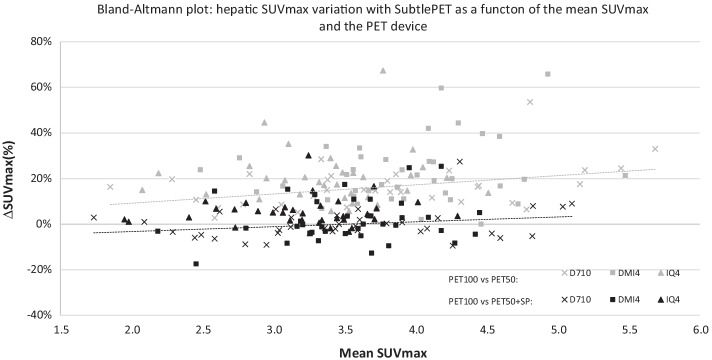
Bland Altman plot of the hepatic SUV_max_ variation (%) as a function of mean SUV_max_ between the 2 PET series, for the 110 patients. The cross, squares and triangles represent the data from the 3 PET devices (D710, DMI4 and IQ4 respectively)

**Fig. 8:**
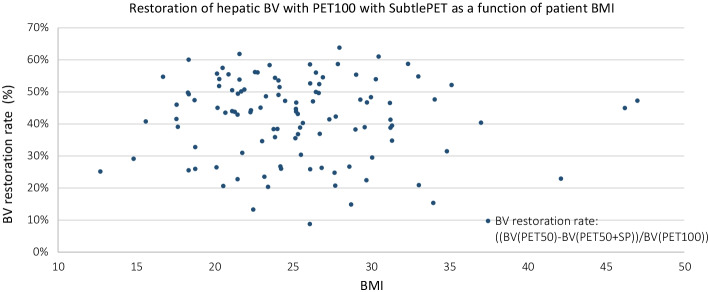
Scatter plot of the restoration of the hepathic Background Variability with respect to PET100 obtained with SubtlePET, as a function of the patient BMI, for the 110 patients

Table 7 and Fig. 7 give the quantitative analyses of the healthy hepatic region for the whole population. Reducing the acquisition statistic by 50% (PET50) logically induced a statistically significant noise increase (*p* < 10^−4^) in comparison with the PET100 acquisition, with a mean (± Stdev) SUV_max_ and a mean (± Stdev) SD going respectively from by 3.4 (± 0.6) and 0.29 (± 0.08) for PET100 to 4.0 (± 0.8) and 0.39 (± 0.09) for PET50. In the SubtlePET-enhanced images (PET50 + SP), the same parameters became comparable, but surprisingly still statistically different to PET100, with mean SUV_max_ (*p* = 0.006) and SD (*p* < 10^−4^) values equal to 3.4 (± 0.7) and 0.28 (± 0.08) respectively. This noise reduction is nevertheless associated with a minor SUV_mean_ increase of 4.1 (± 4.1)%. Considering these 2 last results, we finally observed a slight but statistically significant (*p* < 10^−4^) BV reduction when applying SubtlePET on our PET50 acquisitions (− 7.9 ± 9.9%), as can been seen on Fig. 8. This figure shows that the noise lowering with SubtlePET is independent of the patient BMI.

**Table 7:** SUV_max_, SUV_mean_, standard deviation and background variability quantitative indexes measured on the healty hepatic region on PET100, PET50 and PET50 + SP series of our patients

	PET100	PET50	PET50 + SP	PET100	PET100 + SP
	110 patients			20 patients IMC > 30	
*SUV*_*max*_					
Mean value	3.4	4.0	3.4	4.0	3.6
Standard deviation	0.6	0.8	0.7	0.7	0.6
Mean ΔSUV_max_	–	19.7%	2.3%	–	− 9.6%
Std dev of ΔSUV_max_	–	12.2%	7.9%	–	5.1%
*SUV*_*mean*_					
Mean value	2.2	2.2	2.3	2.6	2.7
Standard deviation	0.4	0.4	0.4	0.4	0.4
Mean ΔSUV_peak_	–	0.2%	4.1%	–	5.0%
Std dev of ΔSUV_peak_	–	1.3%	4.1%	–	2.2%
*SD*					
Mean value	0.29	0.39	0.28	0.35	0.25
Standard deviation	0.08	0.09	0.08	0.08	0.07
Mean ΔSD	–	34.0%	− 4.2%	–	− 28.2%
Std dev of ΔSD	–	12.0%	9.8%	–	11.9%
*BV*					
Mean value	13.1	17.3	12.0	13.5	9.3
Standard deviation	3.2	3.3	3.1	2.5	2.6
Mean ΔBV	–	34%	− 7.9%	–	− 31.5%
Std dev of ΔBV	–	12%	9.9%	–	12.1%

On the right side of the table, the measured for the subgroup of patients with a elevated BMI (> 30)

The analysis of the impact of SubtlePET on the 20 patients with BMI > 30 kg m^−2^ is displayed on the right side of Tables 6 and 7. Regarding those 20 patients, applying SubtlePET on the PET100 images induced a statistically significant noise and SUV_max_ reduction, along with a slight SUV_mean_ increase. The image quality classification (Table 6) was improved for 4/20 exams, from level 2, insufficient quality but interpretable to level 3, image of good quality.

**Fig. 9:**
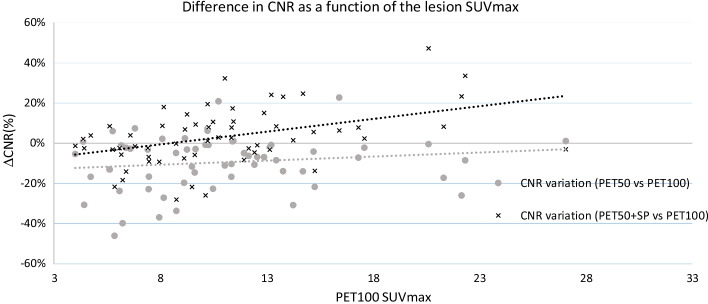
Scatter plot of the variation of Contrast to Noise Ratio between the PET100 and the PET50 ± SP images, as a function of the PET100 SUV_max_, for the 60 lesions selected by the physicians. The CNR restoration is less dependent to lesion SUV_max_ with SubtlePET

Table 8 gives the quantitative analyses of the 60 hypermetabolic lesions selected by the nuclear physicians. In this table, we can observe the overall SUV reduction due to the application of SubtlePET on the PET50 data, in comparison with the PET100 data. For the SUV_max_ index (− 5.4 ± 10.4%) the reduction is statistically significant (*p* = 0.003), although it is not the case for the SUV_peak_ values (− 2.6 ± 5.8%, *p* = 0.15). As previously (Table 7), the noise is reduced when applying SubtlePET. Combining those 2 effects results in a non statistically significant increase (+ 3.3 ± 14.4%, *p* = 0.23) on the lesion CNR which is dependent on the lesion SUV_max_ value as illustrated by Fig. 9. The higher the SUV_max_ on PET100, the higher the CRC increases when applying SubtlePET. On the opposite, the CNR decreases significantly when considering the PET50 data, without the application of SubtlePET (− 9.5 ± 13.6%, *p* = 0.04).

**Table 8:** SUV_max_, SUV_peak_, standard deviation of the background region and Contrast-to-noise ratio indexes measured on the 60 lesions selected by the physicians on PET100, PET50 and PET50 + SP series of our patients

	PET100	PET50	PET50 + SP
	60 lesions		
*SUV*_*max*_			
Mean value	10.9	11.3	10.4
Standard deviation	4.9	5.3	5.3
Mean ΔSUV_max_	–	2.9%	− 5.4%
Std dev of ΔSUV_max_	–	9.2%	10.4%
*SUV*_*peak*_			
Mean value	8.1	8.2	8.0
Standard deviation	4.5	4.6	4.8
Mean ΔSUV_peak_	–	1.2%	− 2.6%
Std dev of ΔSUV_peak_	–	4.4%	5.8%
*SD of the background region*			
Mean value	0.29	0.32	0.27
Standard deviation	0.14	0.15	0.14
Mean ΔSD	–	16.4%	− 8.4%
Std dev of ΔSD	–	15.8%	10.2%
*CNR*			
Mean value	75.2	62.8	83.1
Standard deviation	121.3	86.3	127.0
Mean ΔCNR	–	− 9.5%	3.3%
Std dev of ΔCNR	–	13.6%	14.4%

These results regarding the noise and contrast restoration are confirmed by the qualitative analysis performed by the 2 nuclear physicians. Indeed, target and non-target foci seen on the PET100 images were identically found on the PET50 + SP images, including small sub-centimetric and low contrast lesions. Consequently, they did not find any difference in the PET50 + SP and PET100 image interpretation, and would not have changed their report if it would have been based on PET50 + SP images alone.

## Discussion

This study aimed to evaluate the benefit of the SubtlePET deep learning FDA and CE cleared algorithm, on daily PET/CT images with low count statistic acquisition, especially according to EANM or EU practice guidelines [26]. Only 2 very recent papers evaluated the clinical impact of this Deep Learning approach in low count PET images for more than 60 patients. Our study investigated more precisely the performances of SubtlePET, first from phantom experiments to clinical images of a large population (110 patients) from daily practice, acquired on analogic or SiPM-based PET systems. Our results suggest that SubtlePET clearly allows a good restoration of qualitative (i.e. noise level) and quantitative (i.e. SUV_max_) parameters on PET/CT images with a 50% reduction of total counts according to EU injection guidelines.

In our work, a first series of phantom experiments allows us to evaluate the limits of SubtlePET considering 50% and 33% of the standard statistic. In the first case, the noise level, along with the SUV_max_ indexes are compensated for with SubtlePET (i.e. PET50 + SP). At the opposite, even improved by SubtlePET, our results suggest that a 66% reduction in statistics (i.e. PET33 + SP) leads to a potential loss of information in terms of noise increase and most importantly, lesion detectability for the D710 and DIQ4 PET system. The SiPM-based DMI4 PET system is less limited by the statistic reduction, since for E1 & E2 experiments, the detectability seems to be preserved event with 33% of the statistic. This result was confirmed by the analysis of patient image quality in Table 6, where SubtlePET enhancement of PET33 images did not restore the quality of PET100 images (although for 4 patients, SubtlePET provides good image quality restoration as shown in Additional file 1: Fig. S10). The reason for this can be attributed to the training data and the corresponding noise restoration task. Since all training data was from North America (NA) where the dose of FDG injected is higher (PET100: ~ 6 MBq/kg), the PET25 of NA can be approximated to ~ 1.5 MBq/kg. This dose is still higher than the PET33 of EU which is ~ 1 MBq/kg. Since the SubtlePET algorithm was not trained to restore such low count PET, the resulting performance is not optimal. Considering this main limit of the SubtlePET algorithm for EU, we decided to focus our patient analysis using only a 50% reduction in the statistic. These limitations must however be investigated for each user facility, since they are clearly dependent on the type of PET system and the image acquisition and reconstruction set-up.

The analysis of the image quality by our two senior physicians showed that even for PET100 images, the image quality can be heterogeneous and of poor quality. This can be explained by the presence of patients with a high BMI and by the imaging system used. For example 13/16 level 2 images were acquired on the GE D710 system. It can also be seen that, in the presence of patients with a BMI > 30, applying SubtlePET to PET100 images potentially improves the image quality and therefore the interpretation of the images.

In a previously published paper, Chaudhari et al. report comparable SUV_max_ and SUV_mean_ values between PET100 and PET25 + SP images evaluated on 65 lesions in 15 patients [21]. This is partially in line with our conclusions, since we obtained comparable SUV_max_ values on PET50 + SP or PET33 + SP images, but statistically reduced SUV_mean_ values on the spheres of the phantom experiments and on the patient’s lesion. Most of the differences in quantitative SUV metrics (mean, peak, Max) after SubtlePET processing were ~ 5% of PET100. This variation is clinically insignificant and falls below the test-rest reproducibility of PET scans.

Recently, Schaefferkoetter et al. assessed the performances of a simpler U-Net architecture on patients [19]. The evaluation involved 20 patients (including 12 patients presenting 65 lesions), for whom each image was reconstructed with acquisition times divided by up to 20. The enhanced low time images were compared to the full-time image and the low time smoothed images. The authors noted a noticeable improvement in overall image quality with their deep learning approach, but associated with a degradation in the mean quantification of lesions. The impact on SUV_max_ values was not assessed by the authors.

A very recent paper investigates the ability of SubtlePET to produce images of adequate diagnostic confidence which were considered non-inferior to native scans with two different non-TOF PET/CT scanner models, for a 2/3 FDG standard dose, on 61 patients [24]. They did not investigated a larger dose reduction due to practical considerations, but not from objective reasons. Our results on phantom experiments, clearly confirm that a dose reduction up to 50% can be achieve without any lost in the lesion detectability, signal to noise ratio or quantitation. Our clinical results are in line with this work, since they reported no significant difference between datasets in lesions’detectability, target lesion mean SUV_max_ value, and liver mean SUV_mean_ values. No false-positive lesions were neither reported in PET enhanced with SubtlePET.

Compared to other deep learning based denoising approaches [18, 19], the optimization steps implemented in SubtlePET were able to correctly recover the SUV_max_ values of the lesions maintaining a noise level equivalent to full-time images.

We also limited our study to one lesion per patient in order to remove any bias in the quantitative analysis, using multiple lesions coming from the same reconstruction as it was done by Chaudhari et al. or Schaefferkoetter et al. [19, 21].

Another limitation of our study concerns the use of spheres in the phantom of much smaller sizes than the lesions found in our patients. The issue of detectability really arises for small lesions. Since one aim of this work was to evaluate the limits of SubtlePET, we found it more interesting to integrate microspheres in our phantom experiment. We thus showed that reducing the acquisition time entailed a risk of non-detection of small lesions and that SubtlePET was not able to recover these lesions. This is quite understandable as this approach consists in detecting the hot spots of the images to differentiate noise from useful signal. Since the signal is not distinguishable from the noise present in the images, the software cannot recreate this signal which is somehow expected. In the case of pre-identified small lesions (< 7 mm), a longer acquisition can then be performed.

These results were obtained by considering a reduction in counting by reducing the acquisition time of list-mode data. As the counting statistic varies linearly with the number of coincidences detected, these results can be extrapolated to a direct reduction of the injected dose, at the limit of the random coincidences rate variation [25].

Practically, SubtlePET offers new perspectives such as, dividing the injected dose by 2 to reduce the patient and staff exposure, scanning twice as fast when necessary (children, painful patients, long delays in appointments and the need to increase the number of patients per day, emergencies, etc.…) or the possibility to make real whole body and not mid-thigh vertex more easily. On the other hand, we can question ourselves on the interest to use SubtlePET on low noise images obtained with the latest long FOV SiPM PET models that offer almost noise-free images. Since SubtlePET is designed to denoise PET images, applying it on noise free or PET100 images could result in additional smoothing that would degrade the interpretability of the exams, with a loss of quantification as shown for large patients (Table 7).

In this work, we investigated SubtlePET on F-18-FDG PET images. SubtlePET™ is FDA-cleared for use with 18F-FDG and 18F-Amyloid tracers and, since june 2021, is now CE-marked for use with 18F-FDG, 18F-Amyloid, 18F-Fluciclovine, 18F-DOPA, 18F-Choline, 18F-DCFPyL, Ga-68 Dotatate and Ga-68 PSMA radiotracer PET images, expanding coverage for Prostate (PSMA) and Neuroendocrine tumors. It will be interesting to evaluate the performances of such algorithm on other radioactive tracers such as ^82^Ru for which the image is naturally smoother due to higher energy of the positron and a larger mean free path of the positron. The same approach will have to be extended to other F-18 radiolabeled since their spatial distribution which was potentially learned for the CNN differs from the FDG distribution. In parallel to this study, we tried to test SubtlePET™ 2.0 with other tracers like F-Dopa or F-Choline and the visual results seem to be as effective as those obtained with FDG (Additional file 1: Fig. S11). A similar approach to SubtlePET could also be used for SPECT imaging, either to reduce dose or acquisition time.

## Conclusion

We assessed the performances and potential limitations of a new neural network-based denoising approach on phantom datasets and on a large patient population from daily practice, benefiting from F18-FDG PET exams based on EU recommended standard of 3 MBq/kg injected activity. In our phantom study and patient image quality analysis, reducing the count statistic as low as 33% leads to a loss of detectability and loss of image quality, making the image not good enough for interpretation. Compared to other similar architectures, for the half-duration acquisitions, the optimization steps implemented in SubtlePET statistically recover the quantification of the phantom experiments and correctly but not statistically recover the SUV_max_ values of the lesions. It also maintain a noise level equivalent to full-time images, yielding a lesion contrast-to-noise ratio comparable to that of full statistical PET acquisition and did not induce any modification of the final clinical report. Based on our results, SubtlePET is adapted to clinical practice for half-duration or half-dose acquisitions based on EU practice of 3 MBq/kg without diagnostic confidence loss.

## Supplementary Information

**Additional file 1** **Figure S10** – Examples of MIP attenuation corrected PET images reconstructed with PET100, PET50, PET33, PET50+SP and PET33+SP obtained on the DMI4 PET system. Male, 76 Years old, Lung cancer of the Right Upper Lung with node and bone metastasis. Initial staging. Characteristics: 68 kg, 182 cm, BMI 20,5, FDG PET 3MBq/kg, 1,5 min/bed position. Q1, Q2 and Q3 represent the quality level given by the nuclear physician: Level 1: insufficient quality, interpretation impossible; Level 2: insufficient quality but interpretation possible and Level 3 : image of good quality. **Figure S11** – supporting data– Examples of MIP attenuation corrected PET images reconstructed with PET50 and PET50+SP obtained on the DMI4 PET system. Male; 90 kg; IBM 30,2; Prostate cancer. Initial staging. F-Choline PET. 2 MBq/kg; acquisition time 1.5 min/bed position.

## Data Availability

The data will be made available upon reasonable request.

## Abbreviations

AI: Artificial intelligence
BMI: Body mass index
BPL: Bayesian penalized likelihood
BV: Background variability
CE: Commission Européenne
CNN: Convolutional neural network
CNR: Contrast to noise ratio
CRC: Contrast recovery coefficient
EANM: European association of nuclear medicine
EU: European Union
F-18-FDG: F-18-fluoro-2-deoxy-glucose
FDA: Federal drug administration
IEC: International electrotechnical commission
PET/CT: Positron emission tomography coupled with computed tomography
PET/MR: Positron emission tomography coupled with magnetic resonance imaging
PSNR: Peak signal to noise ratio
NEMA: National electrical manufacturers association
NN: Neural network
OSEM: Ordered subset maximum expectation
SD: Standard deviation
SSIM: Structural similarity
SUV: Standard uptake value
SUV_max_: Maximum value of the standard uptake value
SUV_mean_: Mean value of the standard uptake value
SUV_peak_: Peak value of the standard uptake value
U-net: Encoder–decoder neural network
VOI: Volume of interest
