# Supplementary material for: Clinical and phantom validation of a deep learning based denoising algorithm for F-18-FDG PET images from lower detection counting in comparison with the standard acquisition
Source: EJNMMI Phys. 2022 May 11;9:36. doi: 10.1186/s40658-022-00465-z (PMC9095795; doi:10.1186/s40658-022-00465-z)
Supplement: Supplementary file 1 — Additional file 1 Figure S10 – Examples of MIP attenuation corrected PET images reconstructed with PET100, PET50, PET33, PET50+SP and PET33+SP obtained on the DMI4 PET system. Male, 76 Years old, Lung cancer of the Right Upper Lung with node and bone metastasis. Initial staging. Characteristics: 68 kg, 182 cm, BMI 20,5, FDG PET 3MBq/kg, 1,5 min/bed position. Q1, Q2 and Q3 represent the quality level given by the nuclear physician: Level 1: insufficient quality, interpretation impossible; Level 2: insufficient quality but interpretation possible and Level 3 : image of good quality. Figure S11 – supporting data– Examples of MIP attenuation corrected PET images reconstructed with PET50 and PET50+SP obtained on the DMI4 PET system. Male; 90 kg; IBM 30,2; Prostate cancer. Initial staging. F-Choline PET. 2 MBq/kg; acquisition time 1.5 min/bed position. [file 40658_2022_465_MOESM1_ESM.pdf]

Figure 10

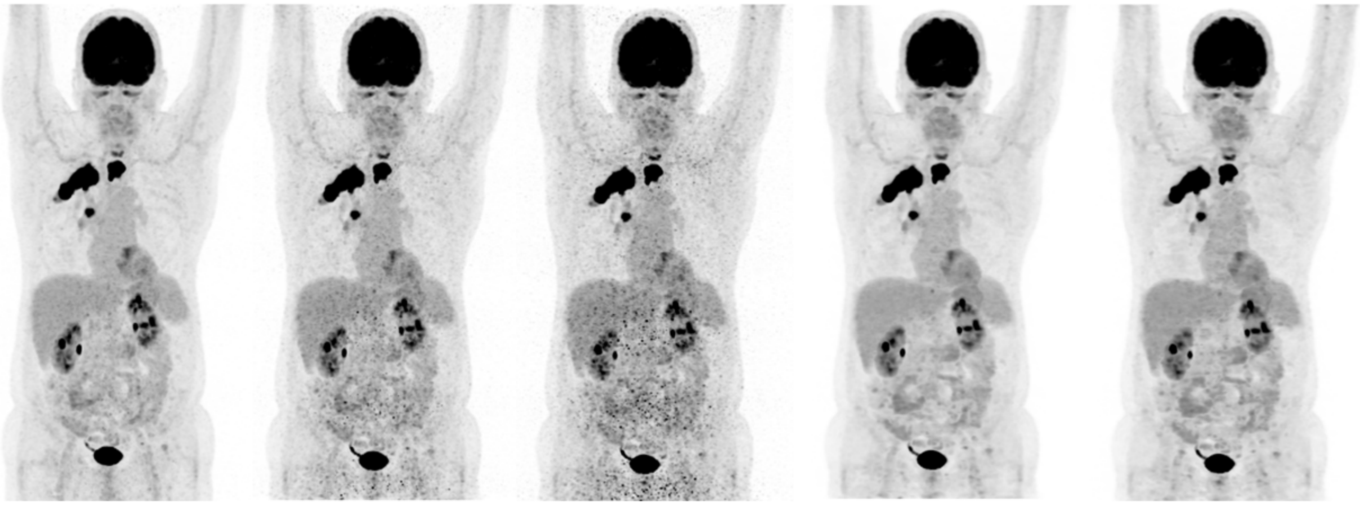

Supplementary file 1 : Figure S10 – Examples of MIP attenuation corrected PET images reconstructed with PET100, PET50, PET33, PET50+SP and PET33+SP obtained on the DMI4 PET system. Male, 76 Years old, Lung cancer of the Right Upper Lung with node and bone metastasis. Initial staging. Characteristics: 68 kg, 182 cm, BMI 20,5, FDG PET 3MBq/kg, 1,5 min/bed position. Q1, Q2 and Q3 represent the quality level given by the nuclear physician : Level 1 : insufficient quality, interpretation impossible; Level 2 : insufficient quality but interpretation possible and Level 3 : image of good quality

Figure 11

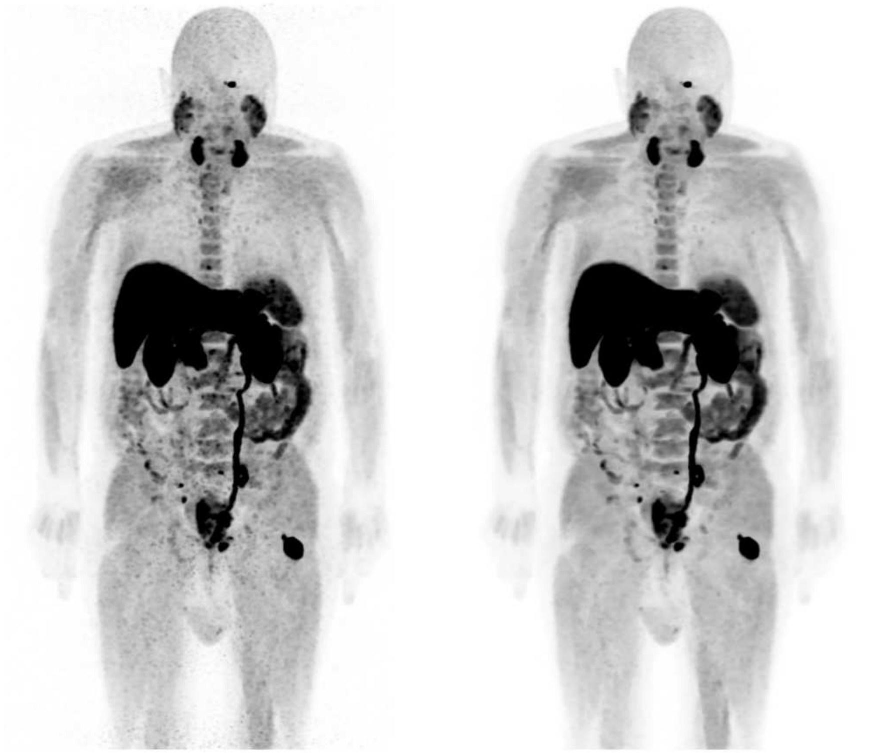

Supplementary file 1 : Figure S11 – supporting data– Examples of MIP attenuation corrected PET images reconstructed with PET50 and PET50+SP obtained on the DMI4 PET system. Male; 90 kg; IBM 30,2; Prostate cancer. Initial staging. F-Choline PET. 2 MBq/kg; acquisition time 1.5 min/bed position
